# Evaluating the nutritional and economic potential of defatted algae cake in aquaculture: A review

**DOI:** 10.1016/j.aninu.2025.11.013

**Published:** 2026-04-29

**Authors:** Aslah Mohamad, Ina Salwany Md Yasin, Amirah-Syafiqah Zamri, Mohamad Azzam-Sayuti, Farhan Nazarudin, Azfar Ismail, Amir-Danial Zahaludin, Liyana Yahya, Fatin-A'lia Bakri, Noorazlenawati Borhan, Chan Wong

**Affiliations:** a Aquatic Animal Health and Therapeutics Laboratory, Institute of Biosciences, Universiti Putra Malaysia, UPM Serdang 43400, Selangor, Malaysia; b China-Asean College of Marine Sciences, Xiamen University Malaysia, Sepang 43900, Selangor, Malaysia; c Department of Aquaculture, Faculty of Agriculture, Universiti Putra Malaysia, UPM Serdang 43400, Selangor, Malaysia; d Department of Veterinary Laboratory Diagnosis, Faculty of Veterinary Medicine, Universiti Putra Malaysia, UPM Serdang 43400, Selangor, Malaysia; e Petronas Research Sdn Bhd, Petronas Research and Scientific, Jln Ayer Hitam, Bangi Government and Private Training Centre Area, Bandar Baru Bangi 43000, Selangor, Malaysia

**Keywords:** Microalgae, Biodiesel by-product, Fish feed, Aquaculture nutrition, Sustainable aquaculture

## Abstract

The increasing demand for sustainable aquafeed ingredients has driven the exploration of alternative protein sources to replace conventional fishmeal and soybean meal. Defatted algae cake (DAC), a by-product of microalgae-based biofuel production, presents a promising and eco-friendly protein source for aquaculture. This review examines the potential of DAC as a sustainable alternative feed ingredient by assessing its nutritional composition, inclusion levels, digestibility, economic feasibility, and environmental benefits. Rich in proteins, essential amino acids, bioactive compounds, and functional lipids, DAC has demonstrated positive effects on fish growth, feed efficiency, and immune responses when incorporated at optimal levels. However, high production cost, variability in nutrient composition, the presence of anti-nutritional factors, and species-specific dietary requirements pose challenges to its widespread application. Comparative analyses indicate that DAC inclusion levels at optimal levels, generally under 10% in fish feed, can maintain growth performance across various species, while higher levels may impact digestibility, feed efficiency, and nutrient bioavailability. The economic viability of DAC is influenced by its cost relative to traditional feed ingredients, advancements in processing, improved digestibility for fish, and integration within circular bioeconomy models. Additionally, its use in aquaculture contributes to sustainability by reducing reliance on overexploited marine resources and mitigating environmental impacts associated with fishmeal and soybean meal production. This review highlights the need for further optimization in DAC processing and formulation to enhance its utilization as a cost-effective and sustainable aquafeed ingredient, supporting the long-term growth of the aquaculture industry.

## 1 Introduction

Aquaculture is the fastest-growing sector in global food production, contributing 51% of the world's food fish supply (FAO, 2024). Inland aquaculture accounts for 62.6% of total farmed aquatic animal production, highlighting the urgent need for sustainable and cost-effective protein sources to support its continued expansion (FAO, 2024). This rapid growth is driven by increasing demand for fish as a high-quality protein source, positioning aquaculture as a critical supplier of nutrient-dense food for human consumption (FAO, 2024). At the same time, aquaculture functions as a livestock production system, requiring substantial inputs of high-protein, mineral-rich feed to sustain the health and growth of farmed aquatic animals. However, major challenges for the future of aquaculture include finding sustainable and scalable sources of feed ingredients to support the growing population of farmed aquatic animals without compromising the availability of fish or crops used for human consumption or livestock feed.

Feed production relies heavily on fishmeal and soybean meal as primary ingredients in feed. However, unsustainable overfishing required for fishmeal production has led to pressure on in-fishery resource, raising concerns and driving the shift toward plant-based protein alternatives like soybean meal, corn gluten meal, wheat gluten meal, and sunflower meal in aquafeed (Mohan et al., 2022). Using terrestrial plant-based protein in feed formulations faces criticism due to concerns about land use competition with food production, slower plant growth, and its contribution to global deforestation, biodiversity loss, and other environmental and social issues in developing countries (OECD/FAO, 2024; Thornton et al., 2023). The limited availability and rising costs of these ingredients makes it difficult to develop sustainable and cost-effective fish feed, highlighting the need for alternative protein sources in aquafeed production.

To address the growing concerns over the sustainability of conventional aquafeed, the feed industry has turned to eco-friendly alternatives that reduce reliance on traditional fishmeal and other non-sustainable ingredients. Microalgae has emerged as a sustainable alternative to conventional fishmeal and terrestrial plants sources for aquafeed. It offers numerous advantages, including high protein content, a well-balanced amino acid profile, and the presence of functional compounds such as polyunsaturated fatty acids (PUFAs), pigments, vitamins, and active polysaccharides, which can enhance immune responses, antioxidant capacity, antibacterial activity, and colouration in aquatic animals (Ma and Hu, 2024).

**Fig. 1:**
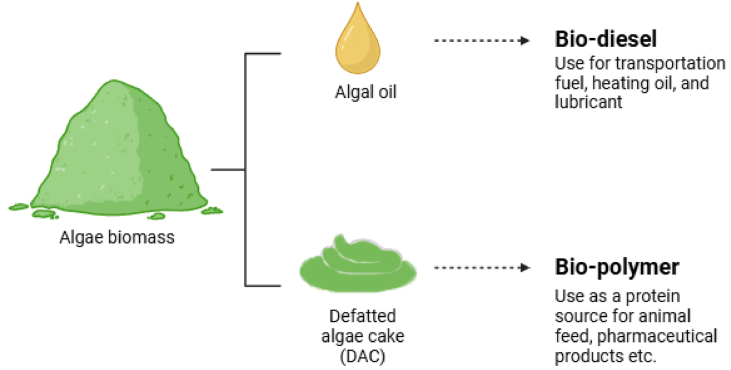
Summary of the products and applications of algae biomass. Adapted from Arun et al. (2022).

Additionally, the production of biofuels from microalgae has attracted significant attention, as the global demand recognizes the ability for biofuels to support the energy transition, ease food pressures, protect the environment, and contribute to climate change mitigation (Wang et al., 2024). Biodiesel remains the only biofuel capable of directly replacing fossil fuels and has been widely cultivated as a lipid-rich feedstock for this purpose (Enamala et al., 2018). In recent years, advancements in microalgae-based biofuel production have also led to the generation of large quantities of a valuable co-product known as defatted algae cake (DAC), a protein-rich by-product derived from microalgae biomass after lipid extraction using chemical (e.g., solvent and supercritical CO_2_), enzymatic, or mechanical (e.g., oil expeller, microwave-, and ultrasonic-assisted) methods (Ahmad Ansari et al., 2020). The downstream processing for biodiesel extraction yields DAC, which, despite the removal of most lipids, retains considerable amounts of other valuable nutrients (Reboleira et al., 2021) (Fig. 1). Due to its nutrient-dense composition and lower environmental footprint, DAC represents a sustainable alternative to conventional protein sources, especially for use in aquafeeds, thus aligning with circular bioeconomy principles by transforming what would otherwise be waste into valuable feed resources, while supporting both energy and food security.

Microalgae protein is particularly promising for the future of sustainable agriculture, as it requires less arable land and water, herbicides, has rapid growth, high lipid content, minimal space requirements, and the ability to capture carbon dioxide gas, which can contribute to zero-carbon initiatives (Fabris et al., 2020; Huang et al., 2022). Moreover, the nutrition profile of microalgae is similar to that of many fish, making it a suitable ingredient for aquafeed (Nagappan et al., 2021). Despite the removal of lipids for biofuel, DAC remains rich in essential nutrients and has shown potential to partially replace conventional protein sources, such as fishmeal, corn, and soybean meal, as fish feed ingredients (Pereira et al., 2020; Valente et al., 2019). Exploring DAC as a feed ingredient offers an eco-friendly solution to reduce dependency on traditional ingredients, ensuring long-term sustainability for the aquaculture industry.

Even though variability in its nutrient composition poses challenges, the inclusion of DAC in fish feed largely depends on its nutritional profile and the need for species-specific formulations to enhance nutrient digestibility and improve feed conversion ratios (Sarker et al., 2025, Sarker et al., 2018). Numerous studies have demonstrated the successful inclusion of DAC in the diets of multiple species of shrimp and fish, which have showed positive effects on growth and feed efficiency at appropriate inclusion levels (Shah et al., 2018). Furthermore, converting a by-product of biofuel production into a valuable ingredient for fish feed supports a circular economy and reduces the aquaculture industry's carbon footprint. This approach minimizes greenhouse gas emissions, decreases land use, water use, and demand for energy resources, and supports the shift toward a zero-carbon economy (Siddik et al., 2024).

This review discusses the potential of DAC from biodiesel by-products as a promising alternative ingredient in fish feed. This study explores the nutritional properties of DAC, thereby highlighting its potential advantages as a microalgae-based feed ingredient. Additionally, this review proposes optimal levels for inclusion of DAC in fish diets, the effectiveness of inclusion of DAC in fish diets across different fish species, and the challenges in utilizing DAC. It also assesses the economic viability and cost-effectiveness of DAC compared to commercial fish feed ingredients, as well as its environmental impact. Ultimately, this study aims to demonstrate how DAC can contribute to the advancement of sustainable aquaculture by offering a more efficient, cost-effective, and environmentally friendly alternative to conventional fish feed ingredients.

## 2 Microalgae as a sustainable feed ingredient

Microalgae are a promising substitute for terrestrial plant-based protein due to their nutritional profile. Studies have shown that incorporating microalgae into aquafeed can enhance fish growth, reproductive performance, and the immune function (Siddik et al., 2024). More than 20 species of algae have been studied, such as *Nannochloropsis oculate* (α-tocopherol), *Arthrospira platensis*, *Phaeodactylum tricornutum*, *Desmodesmus* sp., *Tetraselmis* sp., *Haematococcus pluvialis* (carotenoids and astaxanthin), *Dunaliella salina* (β-carotene), *Chlorella zofingiensis* (astaxanthin and lutein), and *Pavlova* sp. These species are commonly used in aquafeeds due to their excellent nutritional value and suitability for aquaculture applications (Siddik et al., 2024). Research into DAC, a protein-rich byproduct of microalgae following lipid extraction for biofuel production, has gained attention for its potential as a cost-effective protein replacement for conventional feed ingredients (Sarker et al., 2023). Defatted algae cake, despite containing lower lipid levels than whole microalgae, has gained recognition due to its nutritional value, as it is rich in proteins, minerals, carbohydrates, and carotenoids. It contains bioactive compounds and promotes balanced growth and health of fish (Abdelghany et al., 2020). Numerous studies have demonstrated the successful inclusion of DAC in feeds for species like European sea bass (Valente et al., 2019), tilapia (Sarker et al., 2025, Sarker et al., 2018), and shrimp (Gamboa-Delgado and Márquez-Reyes, 2018), showing its potential as a substitute for traditional crop-based protein sources and a cost-effective protein alternative in feed production.

### 2.1 Immunostimulatory and prebiotic effects of microalgae and defatted algal biomass in aquafeed

Despite its nutrient composition, studies have shown that incorporating microalgae into aquafeed can enhance the immune function due to the presence of bioactive compounds in some algae, which have antibacterial properties (phlorotannins, laminarin, phycobiliproteins, and c-phycocyanin) and antifungal effects (butylated hydroxytoluene, amphidinolide, and phycobiliproteins), which can improve fish health (Siddik et al., 2024). Existing bioactive compounds in microalgae can enhance the immune system and serve as a valuable component, reducing reliance on therapeutics and thereby lessening the need for antibiotics and other medications (Valente et al., 2019). Research has shown that even small amounts of *Chlorella* sp., ranging from 0.4% to 1.2%, can improve fish immunity and activate immune-related molecules, such as immunoglobulin M (IgM), immunoglobulin D (IgD), interleukin-22 (IL-22), and chemokines, by regulating their gene expression (Siddik et al., 2024). Similarly, feeding fish with DAC has been shown to have a significant immunomodulatory effect, with studies highlighting thatmoderate levels of DAC inclusion can effectively boost immune function in fish (Valente et al., 2019). Additionally, Reboleira et al. (2021) stated that the defatted biomass of *Aurantiochytrium* sp. presents a valuable resource due to its high protein and dietary fibre content, which acts as a prebiotic, supporting the growth of beneficial gut microbiota, such as *Bifidobacterium*, *Streptococcus*, and *Lactobacillus*. The proliferation of these microbes enhances gastrointestinal health by suppressing harmful bacteria, improving digestive efficiency, and producing short-chain fatty acids that regulate immune responses (Patel et al., 2020; Reboleira et al., 2021).

### 2.2 Nutrient profile and digestibility of defatted algal biomass

Analysis of the nutrient profile of DAC compared with various species of defatted microalgae alongside fish meal and soybean meal (Table 1) revealed that among the microalgae species, defatted *Nannochloropsis* sp. had the highest protein content (429 g/kg), while defatted *Haematococcus pluvialis* and defatted *Tetraselmis* sp. had relatively lower protein values (40.3 and 40.63 g/kg, respectively). According to Sarker et al., 2025, Sarker et al., 2018, although the co-product of *N*. *oculata* contains more protein than whole cells, it is more difficult to digest, making it a less efficient nutrient for absorption by fish than whole cells. These findings explain why higher inclusion levels of DAC result in slower growth than lower inclusion levels in the fish diet. This statement is supported by previous studies, which show that lipid extraction from *N*. *oculata* can increase the levels of anti-nutrients, such as non-starch polysaccharides (NSP), potentially reducing digestibility and growth performance in fish (Sarker et al., 2025, Sarker et al., 2018). However, during extrusion processing, the heat and pressure involved in the process help break down anti-nutritional factors, such as trypsin inhibitors, thereby improving the digestibility of the feed (Sarker et al., 2025, Sarker et al., 2018).

**Table 1:** Comparison of proximate compositions (dry matter) and energy values of various defatted microalgae, soybean meal and fishmeal incorporated into fish feed.

Item	Protein, %	Lipid, %	Ash, %	Energy, kJ/g	References
Soybean meal	42.04	2.00	5.54	17.45	Pereira et al. (2020)
Fishmeal	71.85	6.90	13.07	19.78	Pereira et al. (2020)
Defatted *Nannochloropsis* sp.	45.20	7.60	23.60	18.30	Valente et al. (2019)
Defatted *Haematococcus pluvialis*	38.60	3.40	12.90	17.80	Jiang et al. (2019)
Defatted *Tetraselmis* sp.	40.63	1.29	14.57	17.10	Pereira et al. (2020)
Defatted *Aurantiochytrium* sp.	26.70	2.4	16.90	NA	Reboleira et al. (2021)
Defatted *Schizochytrium* sp.	17.93	8.45	12.83	NA	Xiao et al. (2021)
Defatted *Chlorella* sp. HS2	26.60	0.00	4.30	NA	Yun et al. (2022)
Defatted *Nannochloropsis oculata*	44.26	11.73	11.43	3.24	Sarker et al. (2023)
Defatted *Haematococcus pluvialis*	26.95	6.48	3.65	NA	Ma et al. (2019)
Defatted *Nannochloropsis oculata*	36.78	5.60	4.85	12.00	Sarker et al. (2020)
Defatted *Nannochloropsis* sp. QH25	58.03	0.23	14.57	2.68	Sarker et al., (2020)
Defatted *Scenedesmus obliquus*	40.00	8.60	7.50	12.90	Ahmad Ansari et al. (2020)

NA = not available.

## 3 Microalgae as a sustainable feed ingredient

Microalgae by-products, such as DAC, have gained attention as a sustainable alternative to conventional fish feed ingredients (Ahmad Ansari et al., 2021). Incorporation of DAC into aquafeeds can enhance feed sustainability while supporting fish growth, health, and immune responses. However, establishing optimal inclusion levels is critical to ensure feed safety, palatability, and nutritional adequacy, while preventing adverse effects at higher levels. Species-specific studies demonstrate varying tolerance thresholds. For instance, in Atlantic salmon (*Salmo salar*), diets containing 20% defatted *Desmodesmus* sp. biomass maintained growth and health indicators, although the feed conversion ratio increased (Kiron et al., 2016). In yellow perch (*Perca flavescens*), 10% defatted *Haematococcus pluvialis* successfully replaced 25% of fishmeal without affecting growth, whereas higher replacement levels (50%–75%) significantly reduced performance due to nutrient imbalances (Jiang et al., 2019). Similarly, in gilthead sea bream (*Sparus aurata*), the inclusion of defatted *Nannochloropsis* sp. up to 15% resulted in preserved growth and gut health, with the strongest immune stimulation observed at a 10% inclusion rate (Valente et al., 2019).

These findings demonstrate that DAC can be effectively incorporated into aquafeeds, but its benefits are highly dependent on species-specific requirements and inclusion thresholds. Moderate inclusion levels not only sustain growth performance and feed efficiency but can also enhance immune function; however, excessive inclusion compromise nutrient utilization and health outcomes. Thus, future feed formulations should focus on improving nutrient digestibility and balancing amino acid and fatty acid profiles to enable higher, yet safe, levels of DAC inclusion. Such refinements would strengthen the role of DAC as a practical and sustainable ingredient in aquaculture diets. Table 2 summarizes the reported inclusion levels of defatted microalgae biomass in diets for different aquatic species.

**Table 2:** Examples of defatted algae cake (DAC) inclusion in feed from different microalgae species, dosage, efficiency, and limitation in various aquaculture species.

Item	Inclusion levels, %	Host	Advantages	Limitations	References
*Tetraselmis* sp. CTP4	10	Gilthead seabream (*Sparus aurata*)	Higher digestibility of macronutrients and enhanced stress resistance; reduced dietary inclusion of soybean meal.	NA	Pereira et al. (2020)
*Nannochloropsis* sp.	5–15	European sea bass (*Dicentrarchus labrax*)	The 5% DAC diet showed no significant effects on nutrient digestibility, whole-body composition, nutrient retention, or gut morphology.	The 10% DAC diet increased FCR, whereas the 15% DAC diet suppressed immune-related parameters.	Valente et al. (2019)
*Haematococcus pluvialis*	5–15	Yellow perch (*Perca flavescens*)	Dietary supplementation with 5% DAC can replace 25% of fishmeal (10% of the total diet) without negatively affecting fish growth performance or health status.	The 10% and 15% DAC diets reduced growth performance and caused nutrient imbalances, including deficiencies in amino acids and minerals.	Jiang et al. (2019)
*Schizochytrium* sp.	3–12	Mirror carp (*Cyprinus carpiovar*)	The 3% and 6% DAC diets improved fish health by increasing DHA content, optimizing fatty acid profiles, and maintaining intestinal, cardiovascular, and antioxidant functions.	A 12% DAC diet reduced final body weight, weight gain, and specific growth rate.	Xiao et al. (2021)
*Scenedesmus obliquus*	2.5–10	Nile tilapia (*Oreochromis niloticus*)	The 7.5% DAC diet promoted fish growth, enhanced health status, and maintained appropriate biochemical composition.	High inclusion of DAC (10%) reduced fish growth performance and feed digestibility.	Ahmad Ansari et al. (2020)
*Nannochloropsis oculata*	30	Rainbow trout (*Oncorhynchus mykiss*)	Defatted *N*. *oculata* decreased the utilization of fishmeal and fish oil, while yielding higher protein content compared with the control diet.	Extrusion improves macronutrient digestibility, but excessive temperatures reduce nutritional value, especially methionine.	Sarker et al. (2023)
*Haematococcus pluvialis*	1	Chinese mitten crab (*Eriocheir sinensis*)	Defatted *H*. *pluvialis* meal is a good astaxanthin source that can reduce feed cost, improve coloration, enhance antioxidant status, and improve immune responses in *E*. *sinensis*.	NA	Ma et al. (2019)
*Nannochloropsis oculata*	3–8	Nile tilapia (*Oreochromis niloticus*)	The DAC diet improved growth, nutrient composition, protein digestibility, and economic efficiency compared to the reference diet.	Slightly higher feed cost compared to the reference control diet.	Sarker et al. (2020)
*Nannochloropsis* sp.	4–10	Rainbow trout (*Oncorhynchus mykiss*)	The DAC decreased fishmeal inclusion, maintained growth performance and survival, and reduced feed cost per kilogram of fish production.	NA	Sarker et al., (2020)
QH25					

NA = not available; FCR = feed conversion ratio; DHA = docosahexaenoic acid.

## 4 Challenges in utilizing defatted microalgae in fish feed

Defatted algae cake, a by-product of biofuel production, presents significant potential for sustainable aquaculture. However, several challenges hinder its widespread adoption, including the high cost of production, variability in DAC quality, the presence of anti-nutritional factors (ANFs), regulatory hurdles, market acceptance, and concerns regarding the safety of human consumption of fish fed diets containing DAC. These challenges are summarized in Table 3. Addressing these issues is critical to ensuring DAC's safety, quality, and acceptance as a viable component of fish feed. Comprehensive testing for contaminants, including toxins, heavy metals, and pesticide residues, is crucial to mitigate the associated health risks (Roy-Lachapelle et al., 2017).

**Table 3:** Challenges, implications, and potential solutions for defatted algae cake (DAC)-based diets.

Challenges	Situation	Solution
High production cost	High cultivation cost, large water use, low biomass density, energy-intensive lipid extraction, and higher production cost.	Improve cultivation efficiency, optimize culture conditions, adopt energy-saving extraction, scale up to lower unit cost, and offset cost via biodiesel revenue.
Variable quality	Nutritional variability due to species, cultivation, extraction methods, and storage condition; risks from solvent residues, high moisture, and contamination.	Standardize cultivation with SOPs with strict QA, apply cost-effective drying, and ensure stable storage conditions.
Source of nutrient	Strong odour, dark colour, low stability, digestibility, protein yield, and possible toxin contamination.	Fermentation, purification, and stabilization techniques to improve algae quality. Use bioremediation and genetic engineering to enhance quality and yield.
ANF hurdle	Compounds (e.g., phytic acid, tannins, and lectins) reduce nutrient absorption and growth; possible toxin and heavy metal contamination.	Use enzymatic treatments, select low-ANF species, use extruder for pelletization, and apply moist heat to reduce ANFs.
Regulatory barrier	Extensive testing needed to meet safety/nutritional standards; lack of standardize global regulations.	Develop standardized protocols, conduct rigorous testing, and collaborate with regulators.
Market adoption	Concerns about contamination, biofuel by-product links, and safety of DAC-fed fish for consumption.	Ensure transparent sourcing, provide certified safety analyses, obtain eco-label certification, and strengthen consumer education on sustainability.
Consumer concerns	Risks of contaminant bioaccumulation and differences in nutritional quality.	Conduct toxin/heavy metal testing, nutritional equivalence studies, and comply with food safety standards.
Sustainability	Energy-intensive processing increases carbon footprint, environmental impact, and cost.	Apply low-energy methods, use renewable energy, integrate wastewater systems, and adopt circular strategies.

ANF = anti-nutritional factors; SOPs = standard operating procedures; QA = quality assurance.

### 4.1 High production costs

Despite the promising nutrient profile of microalgae, several limitations hinder their large-scale application in aquafeeds. The cultivation of microalgae is highly dependent on light availability due to their photosynthetic nature, making them vulnerable to diurnal and seasonal changes in sunlight. Moreover, optimal growth often requires low biomass concentrations, which increases the need for extensive water use and cultivation area, thereby escalating production costs (Babu et al., 2022). One of the significant economic constraints in the microalgae value chain is the cost-intensive lipid extraction stage, primarily due to the energy demands associated with biomass dewatering and lipid recovery. This stage contributes significantly to the overall cost of microalgae-derived products (Russell et al., 2022).

For DAC to be adopted as a sustainable and economically viable ingredient in aquafeeds, it must be priced competitively in relation to conventional feed ingredients, such as fishmeal and soybean meal. For instance, economic analyses suggest that microalgae biomass must be priced around 2.65 USD/ kg to effectively replace fishmeal and approximately 0.66 USD/kg to substitute soybean meal in commercial feed formulations (Siddik et al., 2024). In contrast, the production costs of microalgae are considerably higher, often exceeding USD 4 to 5 USD/kg (Lu et al., 2023). In integrated production systems where microalgal protein and renewable fuels are co-produced, the unit price for the protein fraction alone may reach up to 7.27 USD/kg (Lu et al., 2023). This economic disparity presents a significant hurdle for incorporating DAC into aquafeeds. Nonetheless, as a byproduct of biodiesel production, DAC holds potential for cost recovery through the sale of primary biofuel products. Leveraging this co-product strategy could improve the economic feasibility of DAC utilization. However, substantial technological and economic advancements are still required to reduce production costs before microalgae-derived feed ingredients can be widely adopted in commercial aquaculture operations.

### 4.2 Variability in nutritional quality and storage challenges

Another challenge to consider for DAC incorporation in fish feed is the variation in DAC quality due to factors such as algae species, cultivation methods, and biofuel extraction techniques. Algae species differ in their moisture, lipid, protein, and nutrient profiles, which can result in inconsistencies in the nutritional quality of DAC. Additionally, high moisture content in DAC poses storage and transportation challenges, leading to increased logistical costs. Innovations in cost-effective drying techniques and integrated processing approaches between the biofuel and aquaculture industries could optimize DAC for dual purposes, reducing costs while improving quality (Venkata et al., 2020). Defatted algae cake-based diets must exhibit comparable nutritional quality to compete with traditional feed ingredients, particularly in terms of protein content and beneficial fatty acid profiles.

The chemical solvents used during lipid extraction may leave harmful residues, affecting DAC's safety and usability (Roy-Lachapelle et al., 2017). Standardized cultivation and processing methods, alongside robust quality assurance protocols, are necessary to ensure consistency and safety. Characterizing bioactive peptides in DAC can further enhance its nutritional and therapeutic properties (Venkata et al., 2020).

### 4.3 Anti-nutritional factors and digestibility limitations

The presence of ANF in certain algae species complicates the use of DAC in aquafeeds. Compounds such as phytic acid, tannins, saponins, and lectins interfere with nutrient absorption and fish growth (Kokou and Fountoulaki, 2018). Phytic acid, for instance, chelates with divalent and trivalent mineral ions, reducing their bioavailability and adversely affecting growth performance, thyroid function, feed conversion ratio, and feed intake (von Danwitz and Schulz, 2020). Effective mitigation strategies include enzymatic treatments, fermentation, and the selective cultivation of low-ANF algae species. Blending DAC with other feed components can also dilute ANFs, while heat processing can reduce certain compounds, such as trypsin inhibitors; however, this may negatively impact nutritional quality (Venkata et al., 2020).

### 4.4 Palatability and sensory limitations

Poor palatability is a significant bottleneck in incorporating DAC into fish feeds. Higher inclusion rates have been associated with poor sensory properties such as hardness, intense coloration, and altered taste (Kumar et al., 2022). Addressing these sensory challenges is essential to enhancing DAC’s acceptance and increasing its inclusion levels in aquafeeds (Ahmad Ansari et al., 2021).

### 4.5 Contaminant risks, regulatory compliance, and carbon footprints

Algae grown in contaminated environments may bioaccumulate toxins, such as microcystins and heavy metals, posing risks to fish and human health (Amorim et al., 2021). Strategies to mitigate these risks include sourcing algae from controlled environments, implementing stringent protocols for contaminant testing, and utilizing advanced processing methods to remove toxins. Regulatory hurdles remain a significant barrier to DAC adoption (Kokou and Fountoulaki, 2018).

As a biofuel by-product, DAC faces scrutiny over its classification and safety. Regulatory agencies require comprehensive testing to ensure that DAC meets nutritional standards and is free of harmful contaminants (Amorim et al., 2020). However, the absence of harmonized global standards complicates compliance. Establishing standardized safety protocols, conducting rigorous quality testing, and providing evidence of its efficacy are crucial to gaining regulatory approval and fostering industry acceptance (von Danwitz and Schulz, 2020).

Market acceptance also poses challenges as consumers often associate biofuel by-products with waste and potential contamination. Concerns about the bioaccumulation of contaminants in fish fed diets based on DAC must be addressed through transparent communication regarding DAC sourcing, processing, and safety measures (Kumar et al., 2022).

Additionally, the carbon footprint of DAC production should also be taken into consideration. Although microalgae cultivation can capture significant amounts of CO_2_ during growth, the overall environmental performance is strongly influenced by energy-intensive downstream processes, such as harvesting, drying, and extraction.

Despite these challenges, DAC offers significant environmental and economic advantages. Its integration into aquafeeds reduces reliance on traditional feed ingredients like fishmeal and soybean meal, lowering aquacultures ecological footprint (Ahmad Ansari et al., 2021). To maximize sustainability, it is essential to minimize the life cycle impacts of DAC production, including energy use and emissions during processing (Amorim et al., 2020). Collaboration between the biofuel and aquaculture industries can facilitate the integration of DAC into the circular economy, unlocking its potential as a sustainable feed resource (von Danwitz and Schulz, 2020). By addressing these barriers, DAC can emerge as a viable and sustainable alternative in aquaculture, contributing to global food security and environmental conservation.

## 5 Economic and cost-effectiveness analysis

### 5.1 Feed as a major production cost

Aquaculture feed constitutes 40%–75% of aquaculture production costs, making it a critical factor in the economic sustainability of the industry (Ahmad Ansari et al., 2021; Sarker, 2023). With the majority of global aquaculture relying on formulated feeds, the increasing costs of fishmeal and fish oil due to overfishing and environmental concerns have spurred the search for alternative feed ingredients. Microalgae, as primary producers in aquatic ecosystems, provide a sustainable and nutritionally rich alternative to traditional feed components.

### 5.2 Microalgae as sustainable, cost-effective alternatives

In the current aquaculture system, microalgae (either whole cells or defatted biomass after lipid extraction) have partially replaced traditional feed for species such as Nile tilapia (*Oreochromis niloticus*). Research indicates that incorporating *Scenedesmus obliquus* at levels of up to 7.5% of fishmeal into the diet of Nile tilapia can result in significant growth. After 44 d, the body weight of the fish was approximately 85 times their initial value. Furthermore, a live fish weight gain of 1 g was achieved using 1.36 g of whole algae (Ahmad Ansari et al., 2020). These results underscore the efficiency of microalgae-based diets, which involve inputs such as infrastructure (ponds and buildings), electric energy, oxygen supply, and nutrient addition, with the output being high-quality fish product production (Ahmad Ansari et al., 2020).

Microalgae's potential extends beyond nutrition. Their balanced composition of proteins, essential amino acids, PUFAs, carbohydrates, pigments, and bioactive compounds makes them ideal replacements for fishmeal and fish oil. Unlike fishmeal, microalgae lack anti-nutritional factors, reducing production costs while maintaining feed quality. Although the production cost of microalgae whole cells is higher than fishmeal, innovations in cultivation and processing can potentially close the cost gap. The defatted biomass of microalgae, a co-product of the biofuel and nutraceutical industries, has emerged as a potential, cost-effective aquafeed ingredient.

### 5.3 Cost and technological innovations

The production cost of some microalgae, such as *Spirulina* (USD 5/kg) and Chlorella (USD 10/kg), remains high compared to fish meal (USD 2.65/kg). These costs are driven by energy-intensive cultivation, harvesting, and processing methods. However, innovative cultivation techniques, including fermentation and photovoltaic systems, have significantly reduced production costs. For example, fermentation-based cultivation of *S*. *acuminatus* has reduced production costs to USD 1.07/kg, improving its economic feasibility (Ma and Hu, 2024; Pruvost et al., 2019).

Microalgae-based aquafeeds also offer environmental benefits. By reducing reliance on fishmeal and fish oil, they alleviate fishing pressure on wild stocks and lower the ecological footprint of aquaculture. Moreover, industrial microalgal co-products can enhance feed conversion ratios and reduce nutrient loads in aquaculture effluents, contributing to more sustainable practices (Sarker et al., 2025, Sarker et al., 2018)

Looking forward, the economic feasibility of microalgae in aquafeed depends on scaling up production, improving biomass productivity, and optimizing cultivation systems. While microalgae cannot yet compete with plant-based proteins purely on cost, their functional benefits, such as enhanced fish growth, health, and product quality, justify their inclusion in aquafeed, even at low proportions. For example, functional additives derived from microalgae require less than 1% inclusion in feeds but can significantly improve fish health and performance, presenting a strategic entry point for microalgae in the aquafeed market (Ma and Hu, 2024).

As aquaculture continues to expand, the adoption of microalgae as a sustainable and cost-effective feed ingredient is expected to grow, with advancements in biotechnology and economies of scale driving further cost reductions. Microalgae-based feeds hold the potential to transform aquaculture, benefitting both the environment and the industry's economic outlook.

## 6 Sustainability and environmental benefits

### 6.1 Addressing fossil fuel dependency and climate concerns

The rising global energy demand and depletion of fossil-derived crude oil have intensified the search for sustainable alternatives, as conventional fuel combustion contributes significantly to greenhouse gas emissions, climate change, and public health concerns (Escalante et al., 2022). Using microalgae as a source for biofuel production has emerged as a promising breakthrough, offering renewable energy solutions while reducing dependency on fossil fuels. However, the rapid development of these technologies has led to the accumulation of industrial by-product waste, including nutrient-rich microalgal residues (Nagappan et al., 2021). Integrating DAC into the circular bioeconomy provides a transformative approach to addressing these challenges. As a by-product of biofuel production, DAC exemplifies resource efficiency by turning waste into a valuable asset. This approach aligns with the core principles of the circular bioeconomy, which seek to minimize waste generation, conserve resources, and maximize sustainability across industries (Sujanto et al., 2024). Industries can simultaneously reduce their environmental impact by incorporating DAC into innovative applications, such as aquatic animal feeds, and contribute to a more resilient and efficient economic model (Kant Bhatia et al., 2022; Siddik et al., 2024).

### 6.2 DAC in the circular bioeconomy

The circular bioeconomy model associated with DAC promotes a closed-loop system where waste is minimized and resources are recycled. This approach aligns with international climate goals, such as those outlined in the Paris Agreement, by reducing industrial emissions and promoting sustainable practices (Branco-Vieira et al., 2020). The adoption of DAC demonstrates how industrial by-products can be transformed into valuable resources, setting a precedent for other sectors to follow and contributing to a more sustainable and resilient global economy. The circular use of DAC creates economic opportunities across multiple sectors. Integrating DAC into fish feed industry processes fosters job creation, particularly through the development of specialized processing facilities and sustainable supply chains. Furthermore, a stable fish protein supply enhances food security, particularly in regions where malnutrition is prevalent. The adoption of DAC as a feed ingredient also aligns with global sustainable development goals (SDGs), including responsible consumption, sustainable industry, and climate action.

**Fig. 2:**
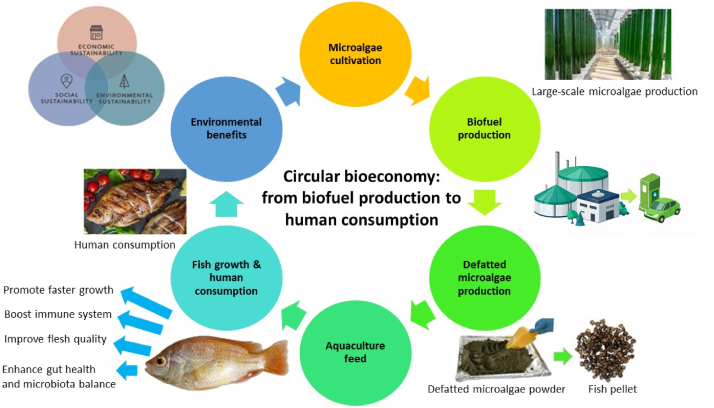
Circular bioeconomy from biofuel production to human consumption via defatted algae cake (DAC) utilization.

Defatted algae cake offers a sustainable solution by repurposing these biofuel residues into products that support economic growth and environmental conservation. The defatted algae are a nutrient-rich by-product, making them an excellent candidate for repurposing in various industries. It contains high levels of protein, essential amino acids, and micronutrients (Kant Bhatia et al., 2022; Pereira et al., 2020; Valente et al., 2019). This composition makes DAC a suitable replacement for traditional feed ingredients, such as fishmeal and soybean meal, which are resource-intensive and environmentally taxing (Fig. 2).

### 6.3 Sustainable alternative to fishmeal and soybean meal

Defatted algae cake represents a sustainable alternative to fishmeal and soybean meal due to its comparable nutritional profile. Incorporating DAC into aquafeeds reduces dependence on wild-caught fish and soybean cultivation, thereby alleviating pressure on marine ecosystems and mitigating deforestation. Diets formulated with DAC have been shown to lower fishmeal and soybean meal usage while maintaining comparable growth, feed conversion ratios, and health outcomes relative to conventional feeds (Gong et al., 2018; Pereira et al., 2020; Shah et al., 2018). For example, rainbow trout fed 10% defatted *Nannochloropsis* sp. achieved growth, feed conversion, and survival rates similar to those of fishmeal-based reference diets (Sarker et al., 2023). Similarly, the inclusion of 10% defatted *Tetraselmis* sp. fully replaced soybean meal without compromising growth performance, while enhancing nutrient digestibility and stress resilience (Pereira et al., 2020). Nutritionally, DAC provides essential amino acids at levels comparable to those of fishmeal and soybean meal (Table 4) and lacks indigestible compounds such as lignin and hemicellulose, which enhances digestibility. It is also a rich source of antioxidants and omega-3 fatty acids, particularly docosahexaenoic acid (DHA) and eicosapentaenoic acid (EPA), making it a suitable replacement for fish oil (Nagappan et al., 2021). This substitution not only supports biodiversity conservation but also contributes to a stable and affordable supply of fish protein for human consumption.

**Table 4:** Comparison of essential and non-essential amino acid concentrations in fishmeal, soybean meal, and various defatted microalgae as protein sources in aquafeed.

Item	Fishmeal, % total amino acids	Soybean meal, % total amino acids	Defatted *Nannochloropsis* sp. *QH25*, g/100 g diet	Defatted *Nannochloropsis* sp., % dry matter	Defatted *Haematococcus pluvialis*, g/100 g	Defatted *Tetraselmis* sp., % fresh matter basis	Defatted *Aurantiochytrium* sp., g/100 g
**Essential amino acids**							
Arginine	3.66	4.55	2.81	3.1	2.58	3.82	1.1
Histidine	1.34	1.64	0.74	0.9	0.81	0.86	1.1
Isoleucine	2.89	3.11	1.60	2.2	1.73	1.72	1.6
Leucine	4.74	5.03	4.02	3.8	3.35	3.16	3.3
Lysine	5.07	4.13	2.66	3.0	2.08	2.16	4.1
Methionine	1.77	0.67	1.06	1.0	0.77	1.10	2.4
Phenylalanine	2.53	3.21	2.37	2.5	2.04	2.79	1.4
Threonine	2.58	2.49	2.59	2.2	2.39	2.10	1.8
Valine	3.38	3.19	2.46	2.8	2.66	1.98	0.93
**Non-essential amino acids**							
Alanine	4.01	2.71	NA	2.9	3.73	2.48	2.2
Aspartic acid + asparagine	5.90	7.26	NA	4.0	3.81	3.27	7.0
Cysteine	0.35	0.59	NA	0.4	0.54	0.37	0.79
Glutamic acid + glutamine	8.96	12.78	NA	4.8	3.73	3.76	18.0
Glycine	4.29	2.76	NA	2.7	2.89	2.28	2.2
Proline	2.18	3.31	NA	2.5	2.08	1.85	NA
Serine	2.26	3.17	NA	2.0	2.00	2.18	4.2
Tyrosine	1.77	1.93	NA	1.7	1.39	2.00	NA
References	Lee et al. (2024)	Lee et al. (2024)	Sarker et al. (2020)	Valente et al. (2019)	Jiang et al. (2019)	Pereira et al. (2020)	Reboleira et al. (2021)

NA = not available.

### 6.4 Conservation of natural resources

Another critical environmental advantage is the conservation of natural resources. The production of DAC requires far less land, water, and energy than traditional feed ingredients such as soybean meal (Nagappan et al., 2021). Soybean cultivation is a major driver of deforestation, particularly in South America (Song et al., 2021), and can only yield 4000 kg/ha per year of protein, compared to microalgae which can yield approximately 366,000 kg/ha per year (Vishwakarma et al., 2022). By replacing soy in aquafeeds with DAC, the demand for agricultural land is lessened, thereby preserving vital ecosystems and reducing the associated carbon footprint. Similarly, fishmeal production, which relies on wild fish stocks, significantly threatens marine biodiversity (Hussain et al., 2024). The defatted algae serve as a sustainable alternative, reducing the exploitation of ocean resources and promoting the health of aquatic ecosystems.

In addition to resource conservation, DAC contributes to improved water quality. Runoff from soybean cultivation introduces excessive nutrients into water bodies, causing eutrophication, algal blooms, and oxygen depletion (Vishwakarma et al., 2022). By contrast, microalgae for DAC production can be cultivated in closed systems such as photobioreactors, which minimize nutrient discharge and deliver a balanced nutrient profile (Kant Bhatia et al., 2022). This approach safeguards aquatic biodiversity and mitigates the negative impacts of agricultural runoff.

Furthermore, DAC reduces the overall carbon footprint of aquafeeds. Conventional practices such as wild-capture fisheries, particularly trawling, are associated with high fuel consumption and carbon emissions, while soybean production emits 0.36 to 0.54 kg carbon dioxide equivalent (CO_2_e) per kg (Lee et al., 2024; Lucić et al., 2025). In contrast, microalgae cultivation sequesters CO_2_ during photosynthesis, and repurposing residual biomass into DAC further offsets environmental costs. Collectively, these advantages establish DAC as a sustainable feed ingredient with a lower life cycle carbon footprint compared to traditional protein sources.

## 7 Risk assessment of defatted microalgae incorporation in aquaculture feeds at different levels

The incorporation of defatted microalgae into aquaculture feeds provides a sustainable alternative to conventional ingredients such as fishmeal and soybean meal. However, the risks associated with its use are strongly dependent on the inclusion level, generally categorized as low (<10%), medium (10%–20%), and high (>20%).

At low inclusion levels (<10%), DAC is typically well-tolerated, posing minimal risks to fish production. Diets containing less than 10% DAC consistently support normal growth, feed intake, and nutrient retention, with high survival rates and no adverse effects on intestinal morphology. Studies using defatted *Tetraselmis* sp., *N*. *gaditana*, and *Schizochytrium* sp. reported no detrimental impacts on growth, survival, or feed conversion ratios. On the contrary, these inclusions improved product quality traits, including omega-3 fatty acid deposition, carotenoid enrichment, and fillet texture (Gong et al., 2018; Pereira et al., 2020; Sales et al., 2021; Xiao et al., 2021). At this range, routine water quality monitoring is generally sufficient, and risks are considered negligible.

At medium inclusion levels (10%–20%), outcomes vary depending on the algal species, host species, and exact inclusion percentage, thereby presenting moderate risks. Diets containing 10%–15% DAC often maintain good growth, feed efficiency, and immune responses. For instance, complete replacement of fishmeal with 10% defatted *Nannochloropsis* sp. in rainbow trout (*Oncorhynchus mykiss*) resulted in growth, feed conversion, and survival comparable to those fed a commercial reference diet (Sarker et al., 2023). However, adverse effects have also been reported. For instance, 12% defatted *Schizochytrium* sp. reduced nutrient absorption and growth in juvenile mirror carp (*Cyprinus carpio*) (Xiao et al., 2021), and 15% inclusion led to elevated feed conversion ratios and suppressed immune parameters in gilthead sea bream (*S*. *aurata*) (Valente et al., 2019). Similarly, diets containing 15%–20% microalgae decreased protein, energy, and dry matter digestibility in Atlantic salmon (*S*. *salar*), resulting in lower growth and feed efficiency (Gong et al., 2019). Although survival generally remains unaffected, careful formulation is needed to maintain palatability and digestibility.

High inclusion levels of microalgae, those exceeding 20%, pose significant risks to fish health, feed performance, and environmental stability. Digestibility challenges can impede nutrient absorption if not treated or processed effectively (Pascon et al., 2021). For instance, Sarker et al. (2023) reported that a high inclusion level of DAC (30%) in fish feed reduced nutrient digestibility, necessitating extrusion processing at elevated temperatures. However, excessively high temperatures were found to decrease nutritional value, particularly regarding methionine content, and could lead to nutrient imbalances (Sarker et al., 2023). Excess undigested matter also deteriorated water quality by fostering bacterial biofilms, elevating ammonia levels, and reducing chlorine efficacy, thereby heightening disease risks (Barger et al., 2020; Solaiman and Micallef, 2021). Effective management requires optimized formulations with amino acid supplementation, biomass pre-treatment, and water quality interventions such as recirculating aquaculture systems (RAS), biofloc technology, or biofilters to recycle excess nutrients and minimize effluent impacts (Kant Bhatia et al., 2022). Without such measures, high inclusion rates contribute to eutrophication, oxygen depletion, and long-term ecological damage, underscoring the need for a careful balance between the sustainability potential of microalgae and the biological and environmental risks associated with excessive dietary inclusion. Table 5 summarizes the risk assessments associated with different inclusion levels of DAC in aquaculture feeds and their broader environmental implications.

**Table 5:** Risk assessment of defatted algae cake (DAC) levels in aquaculture and environmental settings.

DAC levels	Risk		Management	
	Aquaculture setting	Environmental setting	Aquaculture setting	Environmental setting
Low DAC levels (<10%)	No significant risk to fish.	Unlikely to cause significant environmental impact.	Routine monitoring and maintenance.	Limited intervention with continuous monitoring for potential long-term effects.
Medium DAC levels (10%–20%)	May cause some stress or minor health issues in fish; moderate mortality (10%–49%) if not managed properly.	May contribute to increased pollution and disrupt local aquatic ecosystems.	More attentive monitoring and possible interventions like water exchanges or improved filtration are required.	Requires environmental monitoring and possible adjustments to minimize impacts on local ecosystems.
High DAC levels (>20%)	May cause significant stress or mass mortality (50%–100%) if not mitigated; may increase ammonia levels and gives a strong, pungent smell in the water.	May lead to significant environmental issues such as pollution, eutrophication, and disruption of local aquatic life.	Requires rigorous management, including frequent water exchanges, use of advanced filtration systems, and possible use of biofloc technology to mitigate risks.	Requires comprehensive environmental management strategies, including stringent monitoring, and mitigation measures to protect ecosystems.

## 8 Future opportunities and research directions

### 8.1 Technological improvements to enhance DAC's nutritional quality and digestibility

Using microalgae as a replacement for fishmeal and soybean meal in aquaculture presents several challenges, including poor digestibility, limiting nutrient absorption and feed efficiency (Shah et al., 2018). However, advances in processing and formulation are pushing to overcome them. Mechanical methods, such as extrusion, have been proven effective in breaking down complex cell walls and reducing inhibitors, thereby improving protein and lipid availability in species like salmon and tilapia (Gong et al., 2018). More recently, electro-based techniques, such as pulsed electric fields (PEF), have gained attention for their ability to disrupt cell walls while preserving heat-sensitive nutrients, with further refinement needed to improve efficiency and scalability (Machado et al., 2022). Feed formulation strategies also contribute significantly, such as addition of NSP-degrading enzymes, enhancing nutrient utilization and growth (Liang et al., 2022). Studies with *N*. *oceanica* further demonstrate the importance of considering inclusion level, as both low and high dosages can negatively affect protein or lipid digestibility due to resistant cell walls and enzyme inhibitors (Valente et al., 2019). Overall, these findings indicate that overcoming the low digestibility of microalgae in aquafeeds requires a combination of strategies such as effective processing methods, targeted enzyme supplementation, and precise feed formulation. Such strategies enhance nutrient utilization and help realize the potential of microalgae as a sustainable alternative to fishmeal and soybean meal.

### 8.2 The use of heterotrophic cultivation and synthetic biology for continuous microalgae production to mitigate seasonal variability

Microalgae cultivation in open systems is often constrained by seasonal fluctuations in light, temperature, and nutrient availability, resulting in variable biomass yields despite their strong potential for carbon sequestration. Heterotrophic cultivation offers a practical alternative by growing microalgae in fermenters using organic carbon sources, such as glucose or waste-derived sugars, to achieve high-density biomass production under stable, controlled conditions (Zhao et al., 2024). This approach minimizes dependence on environmental factors and reduces overall energy demand. At the same time, advances in synthetic biology through genetic modification of algae lead to improved photosynthetic efficiency, stress tolerance, and metabolic pathways, enabling more resilient strains with higher productivity (Yadav et al., 2025). Together, these approaches strengthen the feasibility of continuous microalgae production, supporting both biofuel and biofuel byproduct developments and broader applications within a sustainable bioeconomy.

### 8.3 Recommendations for further research on optimizing DAC inclusion in different aquaculture systems

Incorporating microalgae into aquaculture diets presents a promising opportunity to enhance sustainability and nutritional quality in fish and shrimp production. However, further research is needed to optimize its use across various aquaculture systems to fully realize its potential. One key area for future research is understanding species-specific responses to the inclusion of microalgae. Different species may exhibit varying reactions to algae-based diets, particularly in terms of growth performance, feed utilization, and nutrient absorption. Research should focus on identifying the optimal inclusion levels for different species, taking into account their unique digestive physiology, nutrient requirements, and tolerance to algae-derived compounds.

Additionally, balancing the nutritional content of microalgae is essential, as they often lack certain amino acids, such as methionine, which are vital for optimal fish growth. Future studies should focus on formulating algae-based diets that address these gaps through supplementation or the development of algae strains with enhanced amino acid profiles. Understanding the ideal nutrient composition of microalgae to meet the specific needs of different aquaculture species will be crucial for ensuring optimal fish health and growth.

Environmental sustainability is another area that warrants further research. While microalgae offer a promising alternative to traditional feed ingredients, such as fishmeal and soybean meal, it is essential to assess the environmental impact of large-scale algae production. Future studies should investigate the carbon footprint, water usage, and overall resource efficiency of algae cultivation, as well as its potential role in mitigating the environmental pressures associated with aquaculture feed production. By investigating these factors, researchers can ensure that microalgae-based feeds enhance the sustainability of the aquaculture industry and contribute to global efforts to mitigate environmental degradation.

Optimizing the inclusion of microalgae in aquaculture systems requires a holistic approach, encompassing species-specific studies, advancements in algae processing techniques, improved nutritional formulations, and assessments of environmental sustainability. By addressing these areas, future research can help unlock the full potential of microalgae as a sustainable and nutritionally beneficial feed source in aquaculture.

## 9 Conclusion

Defatted algae cake exhibits strong potential to be a sustainable alternative to fishmeal and soybean meal in aquaculture, providing both nutritional benefits and a reduced environmental footprint by minimizing reliance on wild-caught fish stocks and large-scale soybean cultivation. Rich in essential amino acids, vitamins, and minerals, DAC can support fish growth and health when included at optimal levels, typically below 10%, though careful formulation is still required to address challenges related to digestibility and anti-nutritional factors. Nevertheless, further research is needed to optimize processing methods, evaluate differences in protein content and microalgae species, and integrate findings through meta-analysis or data synthesis. Additionally, addressing current limitations related to cost-effective production, nutritional optimization, and large-scale integration will be essential. With continued progress, DAC holds significant promise to become a reliable, scalable, and environmentally responsible protein source, making meaningful contributions to the future of sustainable aquaculture.

## Credit Author Statement

**Aslah Mohamad:** Writing – review & editing, Writing – original draft, Visualization, Validation, Investigation, Funding acquisition, Data curation, Conceptualization. **Ina Salwany Md Yasin** Writing – review & editing, Writing – original draft, Validation, Supervision, Methodology, Investigation, Funding acquisition, Data curation, Conceptualization. **Amirah-Syafiqah Zamri:** Writing – review & editing, Conceptualization. **Mohamad Azzam-Sayuti:** Writing – review & editing. **Farhan Nazarudin:** Writing – review & editing. **Azfar Ismail:** Writing – review & editing. **Amir-Danial Zahaludin:** Writing – review & editing. **Liyana Yahya:** Writing – review & editing, Validation, Methodology. **Fatin-A'lia Bakri:** Writing – review & editing, Validation, Methodology. **Noorazlenawati Borhan:** Writing – review & editing, Validation, Methodology. **Chan Wong:** Writing – review & editing, Validation, Methodology.

## Declaration of competing interest

The authors declare the following financial interests/personal relationships, which may be considered as potential competing interests: Liyana Yahya, Fatin A'lia Bakri, Noorazlenawati Borhan, and Chan Wong are currently employed by Petronas Research Sdn. Bhd, Bandar Baru Bangi, Selangor, Malaysia.
